# The Influence of Academic Versus Private Practice Exposure During Urological Training

**DOI:** 10.7759/cureus.107296

**Published:** 2026-04-18

**Authors:** Andrew Lauwagie, Ava Zamani, Nishant Kumar, Sonja Klumpp, Jack Vercnocke, Ana Moser, Aron Liaw

**Affiliations:** 1 Department of Urology, Wayne State University, Detroit, USA; 2 Medical Education, Wayne State University School of Medicine, Detroit, USA; 3 Department of Urology, University of Iowa, Iowa City, USA

**Keywords:** academia, medical education, medical training, private practice, residency, resident perspectives, surgical training, urology

## Abstract

Introduction

While private practice exposure is included in urology residency training, its perceived educational value among residents has not been well characterized. This study aimed to evaluate the potential benefits and drawbacks of training with academic versus private practice physicians, as well as resident satisfaction within each setting.

Methods

An anonymous survey consisting of multiple-choice questions and a free-response section was distributed to all current urology residents in the United States to assess differences in mentorship, autonomy, research opportunities, and clinical and surgical training between academic and private practice settings. Responses were collected from October 2023 through September 2024.

Results

The response rate was 2.8% (50/1810), but only 20 responses met our inclusion criteria. Of note, 70% indicated the same level of mentorship from academic and private practice attendings; 65% indicated more autonomy while working with private practice attendings; 50% indicated similar surgical training from academic and private practice attendings; 50% indicated better clinical skills and knowledge development in academic settings; 75% indicated similarity in the level of respect, fairness, and disciplinary approach from attendings in both environments. Additionally, 95% indicated more research opportunities in academic settings. Notably, multiple free-response comments highlighted a greater level of comfort and less pressure in private practice settings, allowing for better self-perceived performance overall.

Conclusions

Our findings demonstrate that private practice exposure during urology residency enhances autonomy and fosters a low-stress learning environment, supporting its continued integration to enrich resident training and preparedness for independent practice. This highlights its potential value in graduate medical education.

## Introduction

Across American Urological Association-accredited residency programs, many residents rotate through private practice, which has been associated with improved patient communication, satisfaction, and outcomes [1-3]. Despite these benefits, little is known about how residents perceive the value of private practice exposure during training. Therefore, the objective of our study was to evaluate urology residents’ perceptions of training in private practice, specifically examining whether such rotations enhance the residency experience and which aspects benefit the most. In turn, these data may inform those involved in graduate medical education on how to better integrate private practice rotations to optimize resident learning and preparedness for independent practice.

## Materials and methods

We conducted a cross-sectional survey study of urology residents with exposure to both academic and private practice settings as part of their training. The survey was developed by the study team using Qualtrics (Qualtrics, Provo, UT) and included eleven multiple-choice questions and one optional free-response question (provided as online supplementary material). Demographic information collected included year of training, gender, race, and the percentage of training completed to date with private practice attendings.

Survey questions were designed to compare resident perceptions of experiences in academic and private practice settings across domains, including mentorship, autonomy, surgical training, clinical training, research opportunities, and perceived treatment by attending physicians. Responses were recorded as comparative selections (e.g., “more in academic settings,” “more in private practice settings,” or “no difference,” depending on the question). Private practice attendings were defined as physicians not employed by the university or residency program, whereas academic attendings were defined as physicians employed by the university or an affiliated academic institution.

Publicly available email addresses for U.S. urology residency program coordinators were obtained from program websites and used to distribute the Qualtrics survey link, with a request that recipients forward it to current residents. Participation was voluntary and anonymous, as explicitly stated in the survey invitation. Accordingly, we did not ask respondents to identify their residency program. Two follow-up reminder emails were sent to coordinators to maximize response rates; however, direct contact with individual residents was not feasible due to privacy considerations.

Eligibility criteria included current residents at one of the 148 accredited U.S. urology residency programs. Residents without private practice exposure or those at an early stage of training were excluded from analysis. “Too early in training” was defined as interns who were still on general surgery rotations at the time of the survey and had not yet begun urology training.

The primary objective of the study was to compare resident perceptions of autonomy, mentorship, research opportunities, clinical and surgical training, and overall satisfaction between academic and private practice settings. Secondary objectives included qualitative analysis of insights provided through the optional free-response section.

Survey responses were collected from October 2023 to September 2024. Data were exported from Qualtrics for analysis. Demographic characteristics and multiple-choice survey responses were summarized using descriptive statistics and reported as frequencies and percentages. Qualitative responses from the optional free-response question were reviewed and summarized for thematic content. This study was deemed exempt by the Wayne State University Institutional Review Board.

## Results

The survey was emailed to 141 U.S. urology residency program coordinators with publicly available emails for distribution to residents, approximately 1,810 individuals. The response rate was 2.8% (50/1,810). Of the 50 responses received, 20 fit our inclusion criteria (40%, 20/50) and were subsequently included in our analysis. Half the residents were male (50%, 10/20), and 70% (14/20) identified as White/Caucasian. Most were third-year residents or chief residents (55%, 11/20). The demographic characteristics are summarized in Table 1.

**Table 1:** Demographic characteristics of residents reporting perception of training in academic vs. private settings Intern: first year of residency, both general surgery and urology; U1: second year of residency, first full year of urology; U2: third year of residency, second full year of urology; U3: fourth year of residency, third full year of urology; U4/chief: fifth year of residency, fourth full year of urology

Variable	Number	Percentage
Gender		
Male	10/20	50%
Female	9/20	45%
Other	1/20	5%
Race		
White/Caucasian	14/20	70%
Asian/Pacific Islander	5/20	25%
Multiple/other	1/20	5%
Training level		
Intern	4/20	20%
U1	1/20	5%
U2	4/20	20%
U3	6/20	30%
U4/chief	5/20	25%
% Training with private practice attendings		
0-25%	10/20	50%
25-50%	5/20	25%
50-75%	5/20	25%

For perceived mentorship in each setting, 70% (14/20) felt mentorship was similar from attendings in both environments, while 30% (6/20) reported more mentorship from academic attendings. Of note, 65% (13/20) of residents reported greater autonomy with private practice attendings, while 25% (5/20) experienced no difference; 10% (2/20) perceived to have more autonomy with academic attendings. 

**Figure 1:**
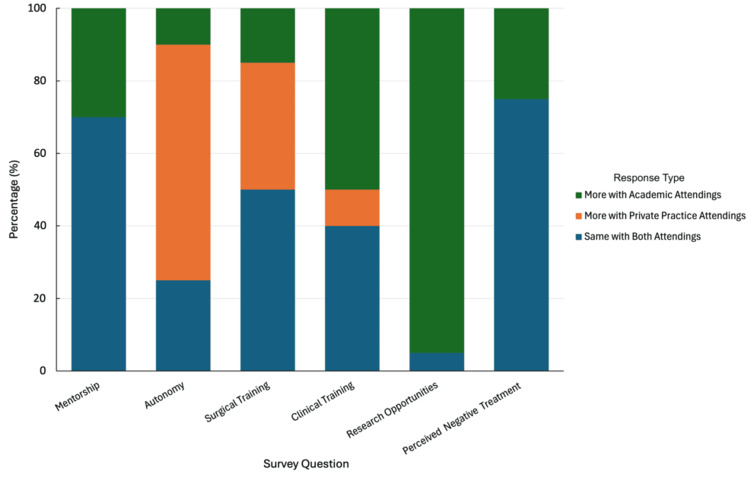
Survey results of resident perception of training in academic vs. private practice settings

Quality of surgical training was viewed similarly in both settings by 50% (10/20) of residents; 35% (7/20) believed private practice offered higher quality surgical training, while 15% (3/20) favored the academic setting. For development of clinical skills, 50% (10/20) reported more development at an academic institution, 40% (8/20) noted no difference, and 10% (2/20) found private practice to offer more to their learning. Notably, 75% (15/20) of residents reported no difference in respect, fairness, or disciplinary approach from attendings in both environments. However, 25% (5/20) felt more negative treatment from academic attendings. Significantly, 95% (19/20) identified academic settings as offering more research opportunities. A summary of the survey results is presented in Figure 1.

Six residents provided additional comments. Two highlighted feeling more comfortable working with private practice attendings, which they felt enhanced learning. One resident elaborated that they felt less judged by private practice attendings, which made them more comfortable asking questions, leading to better surgical performance and expansion of their knowledge base. Another echoed this sentiment, feeling more supported by attendings in private practice, who were more accepting of residents with non-academic interests. This resident felt their academic attendings were annoyed by their lack of academic aspirations. 

Other residents offered more observational insights rather than personal preferences. One mentioned that private practice physicians seemed more valued by their hospital, while another shared that private practice tends to offer higher volumes of quicker cases, whereas academic settings offer fewer, but more complex, cases.

## Discussion

Most residents reported similarities in mentorship, perceived treatment, and surgical training quality across both environments. A smaller proportion of residents perceived more personalized mentorship in an academic setting. Academic institutions have an educational mandate, and their structured focus on teaching and resident development may explain why some residents felt more supported in that environment [4]. Some residents reported experiencing more negative treatment in academic environments. Free-response comments suggested that private practice attendings were perceived as more approachable and less judgmental, creating a more comfortable, low-pressure environment that encouraged questions and supported resident autonomy. One resident noted that private practice physicians appeared to be more valued by their institutions, which, combined with fewer teaching obligations, may foster a more relaxed and supportive atmosphere that enhances learning.

Similar perceptions of treatment are reflected in the literature. Compared with university and military residency programs, residents at community programs are less likely to believe that attendings will think less of them for asking for help. These residents also report higher satisfaction with operative experience and feel that their opinions are valued [5]. Faculty entrustment is a primary driver of resident autonomy, and the supportive culture of non-academic programs has been associated with increased autonomy, greater graduate confidence, and decreased burnout [6-9].

Surgical training quality was not perceived to differ between settings. One resident attributed this to the higher volume of straightforward cases in private practice compared with fewer but more complex cases in academic settings. Differences in case exposure may highlight the complementary value of training in both academic and private practice environments. This is supported by Kim et al., who compared hernia repair approaches between academic and private practice surgeons. Their findings suggest that exposure to both environments may broaden residents’ experience with hernia repair and enhance surgical learning [10]. The lack of difference in surgical training is further supported by evidence demonstrating that surgeons trained in university and non-university settings report similar patient outcomes when practicing in the same clinical environment [11].

For clinical training, higher perceived quality was associated with academic centers, likely reflecting their broader case exposure, structured didactics, and integration of research opportunities. Finally, research opportunities were overwhelmingly seen as more available in academic settings, likely attributable to institutional priorities. Academic centers often include faculty experienced in research, provide dedicated research time, and allocate resources such as statistical and IRB support, all of which are associated with higher resident participation in research [12-16].

Our study has several limitations. The low response rate limits the validity of the results, and the small number of responses meeting the inclusion criteria raises concerns about selection bias, as those who participated may have had particularly strong opinions or experiences. The small sample size and demographically narrow cohort further restrict the generalizability of our findings and the ability to draw definitive conclusions. Additionally, although we defined private practice as physicians not employed by the university or residency program, substantial variability in private practice structure, including hybrid academic-affiliate models, may have blurred this distinction and influenced how respondents interpreted their experiences, potentially affecting internal validity. Finally, while respondent anonymity was intentionally maintained, the inability to identify individual programs reduces our ability to determine whether observed trends reflect national patterns or institution-specific cultures. Future research using larger, more representative samples, clearer definitions of practice settings, and mixed-method approaches incorporating qualitative interviews would provide deeper insight into resident experiences.

## Conclusions

Our findings suggest that private practice exposure during urology residency may be a valuable component of training, particularly in fostering autonomy and creating a supportive learning environment. Incorporating structured private practice rotations into urologic training may improve overall residency experience and better prepare residents for independent practice. Further investigations with larger and more representative samples are warranted to better understand how these training environments can be optimally integrated within graduate medical education.

## Appendices

Supplemental content

*Private Practice vs. Academic Resident Survey*
** **

1. Do you work with Private Practice attendings in your residency? (Private Practice Attending is defined as ‘an attending who does not work directly for the university department/residency program’)

a. Yes

b. No

c. Unsure - with text box 

2. What is your gender? - Selected Choice 

a. Male 

b. Female 

c. Non-binary/third gender

3. What race or ethnicity best describes you? - Selected Choice 

a. White/Caucasian 

b. Asian/Pacific Islander 

c. Multiple Ethnicities/Other

4. What year are you in your residency? 

a. Intern

b. U1 

c. U2 

d. U3 

e. U4/Chief 

5. What percentage of your urologic training to date has been spent working with private practice attendings? 

a. 0-25% 

b. 25-50% 

c. 50-75% 

d. 75-100%

6. How do you compare the level of mentorship available to you in a private practice versus academic setting (i.e., one-on-one teaching, personalized guidance/advice, etc.)?

a. More opportunity for mentorship with private attendings

b. More opportunity for mentorship with academic attendings

c. About the same level of mentorship with private practice and academic attendings

7. How do you perceive your level of autonomy while working in a private practice versus academic settings? (i.e., independent patient care, level of responsibility, opportunities for self-directed learning)? 

a. More autonomous while working in a private practice setting 

b. More autonomous while working in an academic setting 

c. About the same level of autonomy while working in both settings

8. How do you compare the quality of your training as it relates to developing strong surgical skills in a private practice versus an academic setting? 

a. More robust surgical training in a private practice setting 

b. More robust surgical training in an academic setting 

c. About the same quality of surgical training in both settings

9. How do you compare the quality of your training as it relates to developing strong clinical skills and furthering clinical knowledge in a private practice versus an academic setting? 

a. More substantial development of clinical skills/knowledge in a private practice setting 

b. More substantial development of clinical skills/knowledge in an academic setting 

c. About the same quality of clinical training in both settings

10. How do you compare opportunities to get involved with research while working in a private practice versus an academic setting? 

a. More research opportunities available in a private practice setting 

b. More research opportunities available in an academic setting 

c. About the same number of research opportunities are available in both settings

11. In general, do you perceive a difference in the way you are treated by private practice versus academic attendings as it pertains to respect, dismissiveness, fairness, and/or appropriate levels of discipline? 

a. More perceived negative treatment by private practice attendings 

b. More perceived negative treatment by academic attendings 

c. No difference in treatment by private practice and academic attendings

12. Please include any comments regarding your time spent as a urological resident training in private practice and/or academic settings (this may include unique learning opportunities you have had, positive or negative incidents with attending physicians, general attitudes towards the quality of your training, etc.) (All responses are completely anonymous and will be VERY helpful for the purposes of our study. Thank you in advance. *PLEASE DO NOT INCLUDE ANY NAMES OR IDENTIFYING INFORMATION.
